# Metrology of confined flows using wide field nanoparticle velocimetry

**DOI:** 10.1038/srep10128

**Published:** 2015-05-14

**Authors:** Hubert Ranchon, Vincent Picot, Aurélien Bancaud

**Affiliations:** 1 CNRS, LAAS, 7 avenue du colonel Roche, F-31400 Toulouse, France; 2 Univ de Toulouse, LAAS, F-31400 Toulouse, France

## Abstract

The manipulation of fluids in micro/nanofabricated systems opens new avenues to engineer the transport of matter at the molecular level. Yet the number of methods for the *in situ* characterization of fluid flows in shallow channels is limited. Here we establish a simple method called nanoparticle velocimetry distribution analysis (NVDA) that relies on wide field microscopy to measure the flow rate and channel height based on the fitting of particle velocity distributions along and across the flow direction. NVDA is validated by simulations, showing errors in velocity and height determination of less than 1% and 8% respectively, as well as with experiments, in which we monitor the behavior of 200 nm nanoparticles conveyed in channels of ~1.8 μm in height. We then show the relevance of this assay for the characterization of flows in bulging channels, and prove its suitability to characterize the concentration of particles across the channel height in the context of visco-elastic focusing. Our method for rapid and quantitative flow characterization has therefore a broad spectrum of applications in micro/nanofluidics, and a strong potential for the optimization of Lab-on-Chips modules in which engineering of confined transport is necessary.

Nanotechnologies enable the monitoring of steric, electric, and hydrodynamic interactions in confined flows, and open up new horizons in the engineering of new molecular biology assays^1^, the enhancement of osmotic energy conversion^2^, or the development of new fluid functions, including among others fluidic diodes^3^. Accurate flow characterization is most commonly carried out using image-based particle velocimetry^4^. This method, which is based on wide field observation of seeded tracers, has gained popularity due to the simplicity of its implementation^5^,^6^,^7^. It also comes with intrinsic limitations, in particular associated to the thickness of optical sections that makes it difficult to resolve flow profiles in shallow channels of less than ~1–2 μm^8^. Nanovelocimetry techniques using total internal reflection fluorescence (TIRF) imaging, which were pioneered by the groups of Yoda^9^,^10^,^11^,^12^,^13^ and Breuer^14^,^15^,^16^,^17^, take advantage of the exponential decay of excitation energy to determine particle in-axis position. The prevalence of diffusive effects for small tracers, which tend to blur the flow profile, has been successfully addressed by optimizing camera frame rates and developing dedicated image treatment procedures. These technologies now allow for accurate characterizations of near-wall fluid flows, but the thin section of TIRF microscopes of ~200 nm is not adequate to monitor bulk fluid properties in confined flows. Here, we set out to develop an alternative method for accurate *in situ* characterization of confined flows, which merely requires a wide-field fluorescence microscope and conventional 2D tracking algorithms. Flow properties have been characterized based on the analysis of tracer velocity distributions, following a method described in the first section of this report. This method, called Nanoparticle Velocity Distribution Analysis (NVDA) in the article, is then validated using Brownian dynamics simulations of particles flowing in shallow channels. We then show the relevance of NVDA to characterize Newtonian fluid flows in 1.8 μm thick channels, and apply it both to monitor the deformation of shallow channels associated to pressure-driven flows, and to characterize the distribution of particles across the channel height in the context of visco-elastic focusing.

In a shallow channel of 2 μm or less, tracers tracked by wide field fluorescence microscopy remain in focus throughout their migration (Fig. 1A). Their 2D trajectories can be tracked by common tracking algorithms in order to retrieve the longitudinal and lateral velocity distributions along the x- and y-axis respectively (Fig. 1B), which are used as inputs for our analysis. The longitudinal velocity distribution results from the convolution of advection with diffusion along and across streamlines in the x- and z- directions, respectively. According to Faxen law^18^, the velocity of one particle of radius *a* transported in a Poiseuille flow reads:


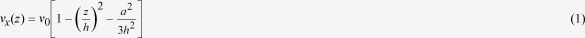


with *v*_*0*_ the maximum fluid velocity, and *z* spanning *–h+a* to *h-a* (left panel of Fig. 1C). Note that hydrodynamic interactions (HI) with the walls are disregarded in the text in order to obtain tractable expressions, but they are considered for fluid flow characterizations using the numerical expressions derived from the work of Pasol *et al.*^19^,^20^,^21^,^22^ (Supplementary Fig. S1). Because transverse migration is disallowed at vanishing Reynolds number in Newtonian fluids^23^, particles are assumed to be homogeneously distributed across the channel height, allowing us to compute the velocity distribution without fluctuations after derivation and reversion of Eq. (1) (middle panel of Fig. 1C). Note that this assumption also imposes the use of high ionic strength buffers in order to screen out wall/tracer electrostatic interaction. Next we consider the contribution of Brownian fluctuations on the velocity distribution. In the simple case of a linear shear flow (shear rate hereafter denoted 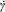), the spreading of the velocity distribution induced by diffusion along the x- and z-axis scales as 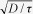 and 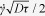, respectively, with *D* the particle diffusion coefficient and 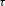 the time interval between two consecutive images. If 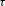 < 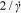, the dominant source of fluctuations is diffusion in the x-direction. With this assumption, we compute the probability that the particle travels at *v*_*x*_:


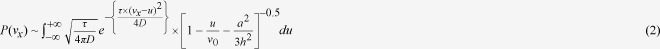


This expression corresponds to the convolution of the diffusion-free velocity distribution with a Gaussian noise. The diffusion coefficient *D* is inhomogeneous in space due to HI, and its amplitude depends on the tracer size, channel height, and fluid viscosity^24^,^25^. Because Brownian fluctuations are isotropic in the x- and y- directions, we propose to consider an “effective” diffusion coefficient *D*_*lat*_, which is extracted from the fitting of the lateral velocity distribution with a Gaussian function (see the green histogram in Fig. 1). This approximation allows us to compute the longitudinal velocity distribution with two adjustable parameters, namely the maximum flow speed *v*_*o*_ and the degree of confinement *a/h* (right panel in Fig. 1C). Consequently NVDA relies on the fitting of longitudinal and lateral velocity distributions to extract the properties of the flow.

We then checked whether NVDA was relevant to measure the flow velocity and channel height. For this we ran running Brownian dynamics simulations of 100 or 200 nm particles conveyed in shallow channels of different heights (see methods section for details on the modeling of advection and diffusion with HI). The resulting longitudinal velocity distributions conformed with the model, taking the inputs of the simulation as fitting parameters (Fig. 2A). We then investigated whether the minimization of the residuals over the parameter space (*v*_*0*_, *h*) between the simulation and our model converged to a unique set of values (Fig. 2B), and then compared them to the inputs of the simulations. This analysis was specifically designed to check the consistency of the main assumptions of the model, namely that (i) vertical diffusion is negligible, and that (ii) longitudinal diffusion is homogeneous across the channel height. Because these approximations are related to the contribution of diffusion, we expect to obtain relevant fits whenever advection is dominant over diffusion. Numerical experiments were thus carried out with increasing Peclet numbers *Pe*, as defined by *U*_*min*_*a/D*_*lat*_ with *U*_*min*_ the minimal velocity of the tracer. We assigned the tracer size to 100 or 200 nm, set the level of confinement and the shear rate 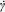 *= 2v*_*0*_*/h* to 0.2 and 1000 s^−1^, respectively, and explored a range of viscosities spanning 0.5 to 16 mPa.s (Fig. 2C). These simulations showed that consistent results were obtained for *Pe* *>* *4* (vertical red line in Fig. 2C). In this regime, the adjustment of numerical experiments was accurate even if 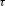 > 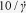 (Supplementary Fig. S2), leading us to conclude that *Pe* was the essential number for NVDA. Finally we wished to assess the precision of the measurement of *v*_*0*_ and *h* as a function of the number *N* of velocity measurements. We observed that relative errors followed a power law scaling with exponent ~-0.5 (black lines in Fig. 2D), and concluded that ~2000 velocity measurements, which could be obtained by tracking ~40 trajectories with our optical settings, were sufficient to characterize *v*_*0*_ and *h* with errors of less than 1% and 8%, respectively.

Next we monitored the transport of neutrally buoyant fluorescent particles (*2a* *= *200 nm) tracers in channels of 1.83{+/-}0.08 μm in thickness and *W* *= *100 μm in width (see fabrication details in Supplementary Fig. S3 and Ref.^26^, and tracer calibration in Fig. S4). Note that the dispersion in height is associated with a difference in etch rate of ~10% between the center and the edge of 10 cm silicon wafers. The viscosity was set to η ~ 5.8 mPa.s using 45% (v:v) glycerin, as inferred from single particle tracking in bulk conditions (not shown). Aqueous fluids of high ionic strength composed of sodium tetraborate (1 mM ; Debye layer *λ*_*D *_~ 7 nm), which is a monovalent salt used in near wall hydrodynamics^11^, or the multivalent buffer TBE 2X (Tris-HCl 160 mM, boric acid 160 mM, EDTA 5 mM, pH *= *8.3; *λ*_*D*_ ~1 nm), were conveyed by pressure actuation in order to minimize the response time to reach steady flows^27^. In both ionic conditions, particle/wall electrostratic interactions were screened over distances much smaller than the particle diameter. Tracers were diluted at a volume fraction of 10^−5^ in order to disregard inter-particle interactions. They were tracked by wide field fluorescence microscopy using the cropped sensor mode of an Electron-Multiplied CCD camera in order to combine high sensitivity and fast temporal resolution^28^. Acquisitions were recorded at inter-frame intervals of 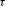 ~ 6 ms with frames of 200 × 400 pixels^2^, equivalently ~20 × 40 μm^2^.

We first analyzed the lateral velocity distribution and extracted the diffusion coefficient *D*_*lat*_ (right panel of Fig. 3A). *D*_*lat*_ represents the diffusion coefficient spatially averaged over the channel height, and its amplitude should be reduced as the thickness of the channel decreases due to HI^24^. This trend was confirmed in the curve of *D*_*lat*_ *vs*. *a/h*, which was in quantitative agreement with analytical models of Brownian motion in confinement (dashed line in left panel of Fig. 3A). For a range of shear rates spanning 100-700 s^-1^ and for mono- or multivalent-salt conditions, the longitudinal velocity distribution was subsequently analyzed with our model (Fig. 3B,C). The channel height deduced from the fit was 1900 nm, and given that ~10000 velocity measurements were considered in the distribution, we estimated the error at ~8% (Fig. 2D). Hence the channel height of 1900 {+/-} 80 nm was consistent with our estimate of mechanical profilometry. Moreover the variation of the velocity *v*_*0*_ with the pressure drop was consistent with Poiseuille law for viscous fluids (Fig. 3D), reinforcing the reliability of our method. Notably because thermal and/or pressure treatments are performed to assemble fluidic devices, the accuracy of channel structural characterization, which is generally carried out before sealing, can be called into question unless they are complemented by *in situ* measurements, as proposed in this report with our NVDA method.

Then we aimed to demonstrate the relevance of NVDA in the context of pressure-driven flows in deformable channels. For this we constructed shallow channels of *2h *~ 2 μm in height and *W*~60 μm in width using the elastomer Poly-dimethylsiloxane of Young modulus *G *~ 1 MPa^29^. Fluid flows were actuated by a constant pressure difference of 30 mbar together with a variable offset *P*_*offset*_ of 100, 300, 500 and 700 mbar in order to trigger increasing levels of deformation. Using scaling predictions^29^, the typical onset in channel height is expected to be *W.P*_*offset*_*/G*~3 μm for *P*_*offset*_ = 500 mbar. The deformation was first characterized using fluorescein with a low numerical aperture objective (100X, NA = 0.6; Fig. 4A), because fluorescence intensity is proportional to the channel height (see e.g. ref. 29). We then rinsed the channel and seeded fluorescent particles. Given the heterogeneity of the flow field across the channel width, we segmented it into 20 sections of 3 μm, which were indexed with the parameter *j* (red dashed lines in Fig. 4B). For each section, we extracted the velocity distribution (Fig. 4B), and analyzed it with NVDA to extract *v*_*0*_^*j*^. Note that we did not estimate the channel height in the fitting procedure, because this parameter was not precisely assessed for strongly deformed channels (a/h~0.05<<1). As the offset pressure increased, we noticed an acceleration of the flow, which was maximal and symmetric about the channel centerline (Fig. 4B, left panel). We then wished to estimate the channel height, and used Poiseuille law to relate it to the maximum flow velocity in each section *j*:


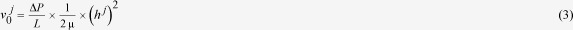


After normalization of Eq. (3) to the velocity measured for j *= *1 at *P*_*offset*_ *= *0 mbar (datasets in Fig. 4C), we inverted Eq. (3) and deduced the relative variations of 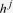, which compared well with fluorescein intensity ratiometric measurements (gray lines in Fig. 4C). Given the channel height and maximum velocity in each section, we finally evaluated the 6-fold onset in flow rate from 8{+/-}1 nL/min to 50{+/-}5 nL/min for offset pressures of 0 and 700 mbar, respectively, with the same pressure difference of 30 mbar. NVDA is therefore available to determine the hydrodynamic resistance in bulging channels made out of polymeric material.

So far we have considered the case of Newtonian fluid flows, in which the repartition of tracers across the channel height is uniform. Focusing of particles toward the channel centerline has been shown to occur in visco-elastic fluids^30^, for inertial flows^31^, and for anisotropic or deformable objects advected in confined channels^32^. Controlling the forces that drive transverse migration may have a strong impact in analytical sciences, in particular for matrix-free separation^33^. Yet most techniques aiming to monitor distributions of tracers across the channel height are only adapted to thick channels of 20 μm or more, and involve long acquisitions based on z-stacks across the flow direction^30^,^34^. We thus propose to adapt NVDA to evaluate the concentration of particles across shallow channels. In order to establish this assay, we first ran Brownian dynamics simulations of 200 nm particles flowing in a 1.6 μm channel according to Pasol model (see methods), and undergoing a transverse force equal to 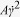 pointing to the centerline with *A* set to 4.10^–19^ Ns^2^. This model is phenomenological, but the expression of transverse forces is consistent with linear models of visco-elasticity^35^. We extracted the longitudinal velocity distribution (blue histogram in Fig. 5A), and observed a peaked shape toward high velocity states in comparison to the prediction of the model with a uniform density of particles (black line in Fig. 5A). We hypothesized that frequent high-velocity events were associated to the accumulation of tracers at the channel centerline, and that depletion from the walls reduced the occurrence of low-velocity events. Therefore the ratio of the experimental velocity distribution to that predicted by the model (that is, with tracers evenly dispersed over the channel height) appeared to be proportional to the vertical repartition of particles. We computed the repartition of particles derived from the velocimetry distribution (datasets in Fig. 5B), and compared it to that directly inferred from the simulation (solid lines in Fig. 5B). The excellent consistency of these two curves showed that NVDA could be used to measure particle spatial distribution across a Poiseuille flow in a confined channel. We then investigated the transport of *2a* *= *100 nm and 200 nm particles in a visco-elastic fluid composed of 2% (m/vol) poly-vinylpyrrolidone (340 kDa) in 2X TBE buffer and channel height of *2h* *= *1.6 μm. We evaluated the maximal flow velocity with NVDA (Fig. 5C), and observed that *v*_*0*_ increased linearly with the pressure drop (inset in Fig. 5C). This result was consistent with our Couette viscometer measurements, showing the constant viscosity with the shear rate, and with earlier reports^36^. Furthermore the viscosity deduced from the linear fit of 5.5{+/-}0.2 cP of NVDA data was comparable to rheological data of 5.8{+/-}0.1 cP (Supplementary Fig. S4). The shape of velocity distributions was subsequently investigated, indicating that 200 nm, but not 100 nm, particles were depleted from the walls (Fig. 5D,E). For 200 nm tracers, depletion from the walls was marginal for shear rates lower than 500 s^-1^ (Fig. 5D), and particles appeared to accumulate away from the middle of the channel at 500 nm from the walls for the maximal shear rate of 2000 s^-1^ (red dataset in Fig. 5D). Due to this bimodal repartition, which showed that particles were repelled from channel centerline where the shear was null, the relative difference in average velocity between 100 and 200 nm tracers *Δv/v*_*0*_ decreased from 9% to 4% for a shear rate of 1000 s^-1^ and 2000 s^-1^, respectively. This result suggested that efficient separation conditions of 100 and 200 nm particles should be obtained at 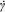
~1000 s^-1^. Altogether NVDA is a versatile tool for the characterization of inhomogeneous concentrations of flowing particles through shallow channels.

In conclusion, We constructed, implemented, and proved the usefulness of NVDA for confined flow characterization. It enables us to monitor bulk hydrodynamic flows whereas evanescent wave-based velocimetry is more relevant to probe near-wall hydrodynamic interactions. While our study provides detailed investigations on channels of 1.5-2 μm in thickness, the characterization of channels of less than 0.5 µm remains to be investigated. Velocimetry in these geometries requires the use of smaller probes of 50 or 100 nm in diameter. These nano-objects can be tracked with the optical system described in this study (not shown), but their diffusion coefficient is greater, and viscosity should be increased to reach adequate conditions in terms of *Pe.* More precisely, we expect *Pe* to scale as:


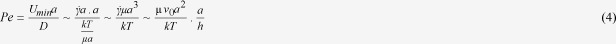


For a given level of confinement, the use of a tracer of 50 *vs.* 200 nm requires the use of a solution 4 times more viscous. Such conditions are accessible to water-glycerol mixtures, but the increased hydrodynamic resistance should be compensated for with shorter channels of ~100 μm. NVDA should then allow us to revisit the transport of spherical tracers under high levels of confinement, in which anomalous^37^,^38^ or normal^39^ responses have been detected near the walls. Specifically, speculations about an apparent slip caused by “molecular behavior in the fluid near the wall”^37^, which is consonant with the debated non Newtonian properties of water near surfaces see e.g.^40^, would need to be clarified. In another direction, Newtonian fluids conveyed in the creeping flow regime have mostly been used in the field of microfluidics, but the recent success of separation techniques involving inertia^41^, complex fluids^42^, or combined inertio-elastic properties^43^ have fostered developments to improve the performances of Lab-on-Chips. Enhanced solute-surface interactions in confined channels may lead to further optimizations, but rapid and quantitative methods for transport characterization are required for engineering fluid flows at these length scales.

## METHODS

### Brownian Dynamics Simulations

We developed Brownian dynamics simulations for a spherical tracer in a confined channel, including the effect of HI with the walls. For Brownian diffusion, we used the approach developed by Ermak and McCammon^44^ with a force field in the z-direction whenever necessary^45^:


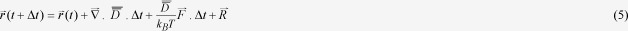


where 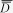 is the tensor of the diffusion coefficient:


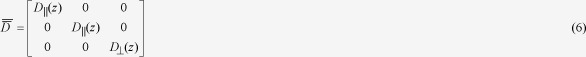


with the subscripts 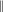 and 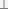 indicating the direction parallel and perpendicular to the walls, respectively. The vector 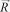 describes Brownian fluctuations:


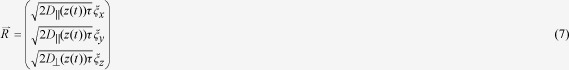


with 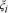 random numbers satisfying 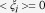 and 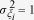. We then included the effect of advection and obtained the final set of equations:


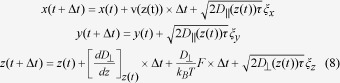


Next we defined the parameters of the simulation. The velocity field was determined by the model described in Pasol *et al.*^21^. The diffusion coefficient was computed using the model of Benesch *et al.*^24^. The time step 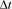 of the simulation should be longer than the time scale associated to inertia 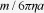
~1 ns given that the density of 200 nm polystyrene particles is 1050 kg/m^3^ and the viscosity is 5 mPa.s. The diffusion coefficient should also remain nearly constant during one time step 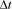 of simulation^12^, and we chose 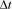 *= *50 and 10 μs for 200 and 100 nm tracers respectively. This time step corresponds to displacements of ~
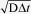
~2 nm for a diffusion coefficient of 0.5 μm^2^/s, and 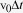
~15 nm for advection for a flow velocity of 300 μm/s. We note that the no-flux condition in the z-direction is satisfied because the diffusion coefficient 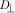 tends to 0 near the walls. The diffusion coefficient derived by the method of Benesch *et al.*^24^ is an expansion, and its computation for arbitrary values of *z* at each time step of the dynamics would dramatically slow down simulations. Hence we computed 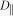, 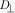, and 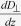 numerically before the simulation with a sampling of 1 nm, and interpolated the exact value of these functions at each time step of the simulation.

The inputs of the simulation were the channel height, the particle diameter, the solvent viscosity, and the flow velocity. We also reproduced imaging conditions by tuning the time interval between consecutive images τ, which was decomposed in an exposure time 6.5 ms followed by charge transfer during 0.5 ms. During the exposure phase, we collected the particle position every 50 or 10 μs, and measured the mean position. The velocity was defined by the difference in mean position between two consecutive images.

## Reagents and Imaging

Chemicals were purchased from Sigma-Aldrich. Nanoparticles were obtained from BangsLabs, and elastomeric channels were fabricated with Sylgard-184 cured during 3 hours at 70 °C. Imaging was performed with a Zeiss epifluorescence microscope equipped with the 38HE filter set (Zeiss), and with a Lumencor Light Engine emitting at 475 nm with a 28 nm bandwidth. An ANDOR iXon-885 camera was used with a binning of 2*2, and a pixel size of 103 nm. Pressure was monitored with a Fluigent Flowell controller delivering 1 bar.

## Author Contributions

H.R., V.P., A.B. wrote the main manuscript text, prepared the figures, and reviewed the manuscript.

## Additional Information

**How to cite this article**: Ranchon, H. *et al.* Metrology of confined flows using wide field nanoparticle velocimetry. *Sci. Rep.* **5**, 10128; doi: 10.1038/srep10128 (2015).

## Supplementary Material

Supplementary Information

## Figures and Tables

**Figure 1:**
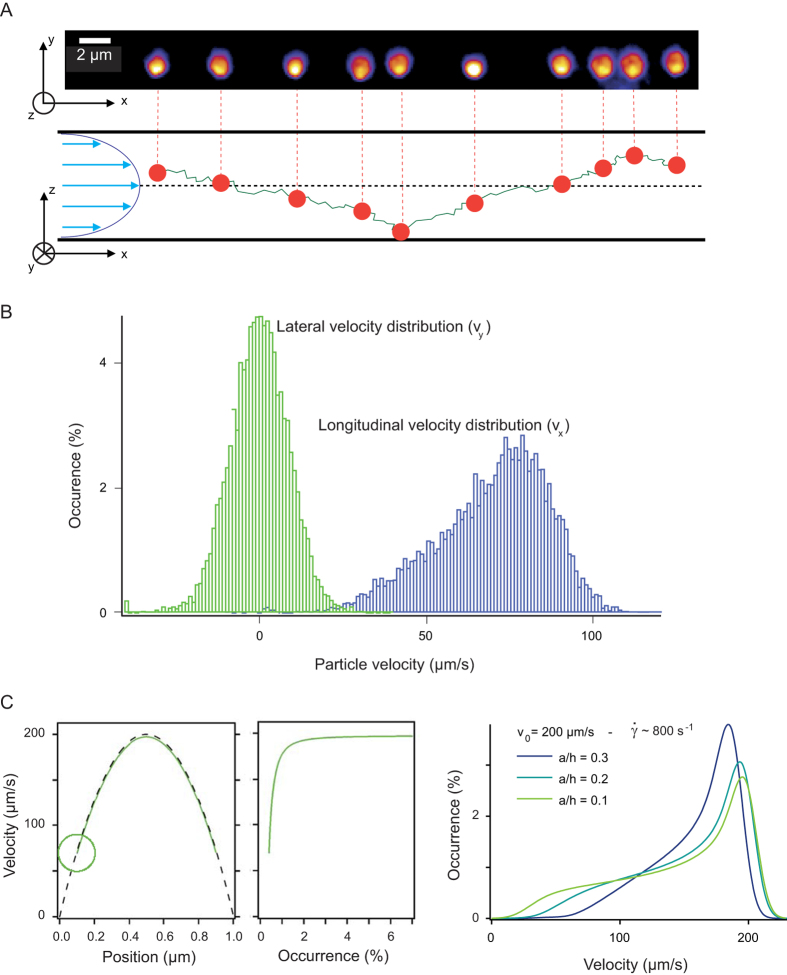
NVDA for confined flow characterization. **(A)** The fluorescence micrograph in the upper panel shows the consecutive position every 35 ms of a 200 nm nanoparticle transported in a 1.6 μm channel. Note that the image has been blurred with a Gaussian filter. The lower scheme represents an hypothetical trajectory across the channel height. **(B)** Typical velocity distributions measured along the x- and y-axes are constructed from typically 5000 events, or equivalently **~**60 particle trajectories. **(C)** The plot in the left panel represents the velocity profile along the z-direction derived from Faxén model for a tracer of diameter a *= *100 nm in channels of height *2h* *= *1 μm transported at *v*_*0*_ *= *200 μm/s (Eq. (1)). Assuming that tracers are homogeneously distributed across the channel height, we compute the velocity distribution (middle panel), and convolve it with an effective longitudinal noise inferred from the lateral velocity distribution (green histogram in (B)). The resulting distribution is computed with two adjustable parameters, namely the maximum flow velocity *v*_*0*_ and the level of confinement *a/h*, which is set to 0.3, 0.2, and 0.1 (blue, cyan, and green curves in the right panel, respectively).

**Figure 2:**
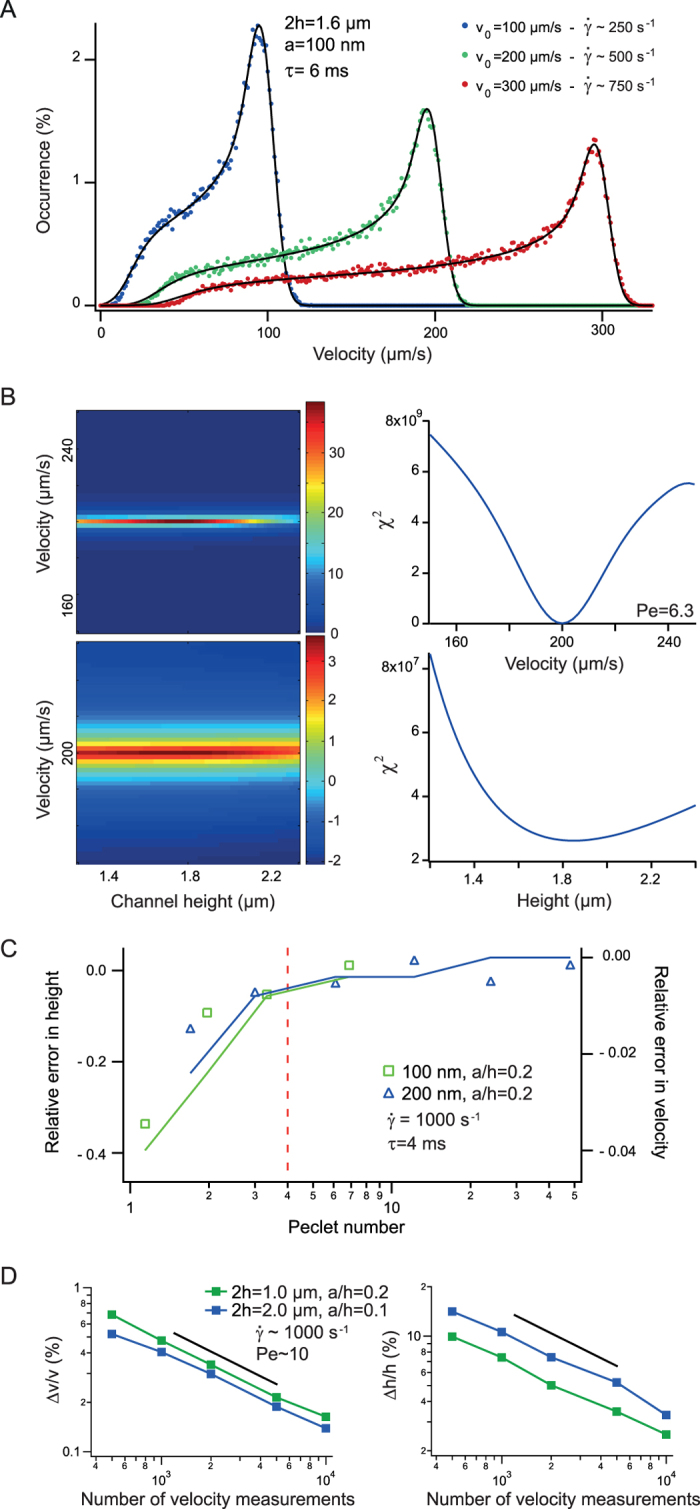
Accuracy of NVDA checked by Brownian dynamics simulations. **(A)** The scattered data points correspond to longitudinal velocity distributions obtained from Brownian dynamics simulations, which are run with 200 nm particles flowing in a 1.6 μm-thick channel at three different flow speeds. Bold lines correspond to the distributions predicted by the model using as inputs the parameters of the simulations. **(B)** The heat maps show the inverse of the residuals *χ*^*-2*^ and its logarithm obtained by fitting the distribution extracted from the simulation at *v*_*0*_ *= *200 μm/s and *2h* *= *1.8 μm with our the model over the parameter space (*v*_*0*_,*h*). The linear plots on the right represent the residuals *χ*^*2*^as a function of *v*_*0*_ or *h*. **(C)** Brownian dynamics simulations are run with 100 nm or 200 nm tracers at a given level of confinement of 0.2 and for a shear rate of 1000 s^-1^. The viscosity of the solution is varied between 0.5 to 16 mPa.s in order to tune the Peclet number (see definition in the text). The relative errors between the fitted values of *v*_*0*_ and *h* and the parameters of the simulation are represented as a function of *Pe* (line or symbol plot, respectively), indicating that precise measurements are obtained for *Pe *> 4. **(D)** The plots show the relative errors on *v*_*0*_ and *h* fitted values as a function of the number of measurements in the distribution. For each data point, we carried out 30 numerical experiments, which were independently fitted to estimate the error of the procedure. The solid trend line is the power law scaling *N*^*-0.5*^ with *N* the number of velocity measurements.

**Figure 3:**
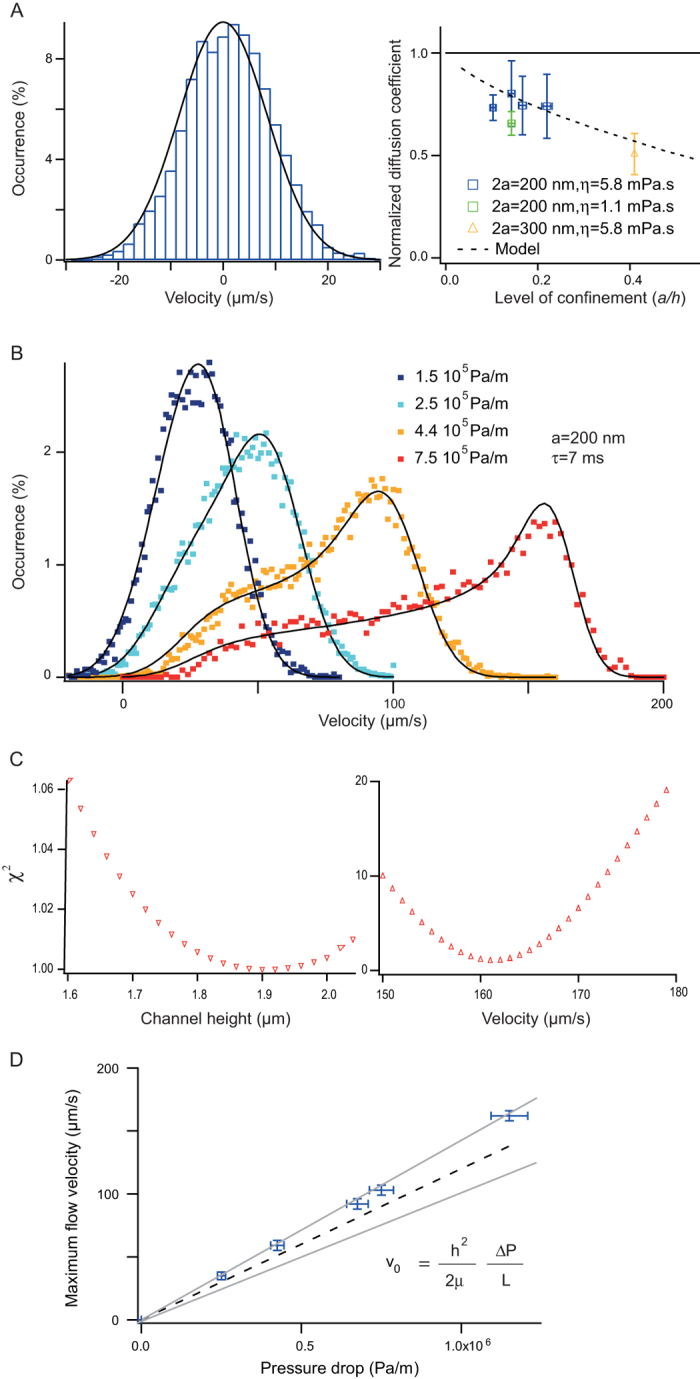
Confined flow characterization with NVDA. **(A)** The lateral velocity distribution obtained for a 200 nm particles conveyed in a 1.83 μm-thick channel is plotted and fitted with a Gaussian function on the right. The diffusion coefficient is hindered by the walls following the predictions of the single-wall reflection method^24^. **(B)** The four velocity distributions are obtained by tracking 200 nm particles flowing in 1.83 μm thickness channels, and the solid lines are the corresponding fits. **(C)** The plots show the residuals for the pressure drop of 7.5 10^5^ Pa/m, defining *v*_*0*_ *= *162{+/-}1 μm/s and *2h* *= *1900{+/-}80 nm. Note that *Pe* was equal to 6 for this analysis. **(D)** The maximum flow velocity increases linearly with the pressure drop (blue dataset), as expected for Newtonian fluids. The prediction of Poiseuille law (dashed line) considering errors in channel length and channel height (gray lines) is consistent with our *in situ* measurements.

**Figure 4:**
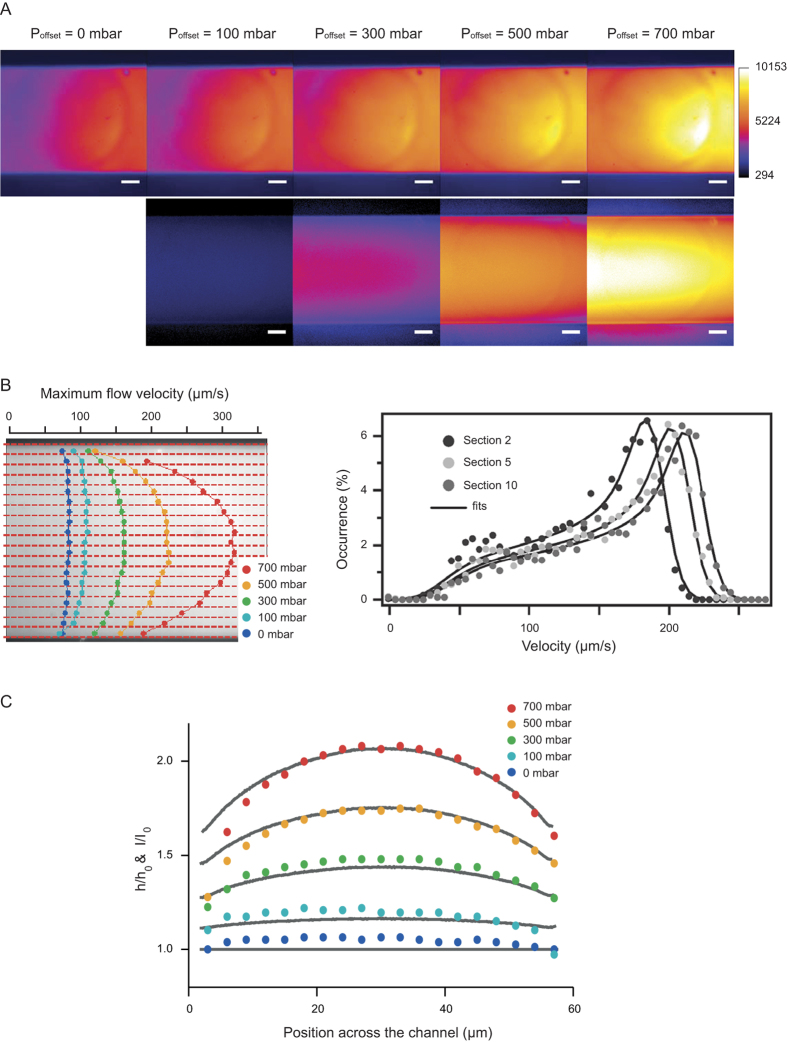
*In situ* characterization of flows in deformable channels. **(A)** The upper five images represent fluorescence micrographs of fluorescein conveyed in 2 μm-thick elastomeric channels. The flow is triggered by a constant pressure difference between the inlet and outlet of 30 mbar with an offset *P*_*offset*_ of 0, 100, 300, 500, 700 mbar. The lower four panels show ratiometric intensity micrographs using *P*_*offset*_ *= *0 mbar as reference. **(B)** The velocity distribution for 200 nm tracers was extracted in 20 sections of 3 μm along the channel width, as indicated by red dashed lines on the left panel. The plot in the right panel represents the velocity distributions in section #2, 5, and 10 at *P*_*offset*_ *= *500 mbar. **(C)** The colored datasets correspond to the variation in channel height along the channel width, as inferred from NVDA (see Eq. (3)), and the associated gray lines are obtained by ratiometric measurements of fluorescein intensity in the panel (**A**).

**Figure 5:**
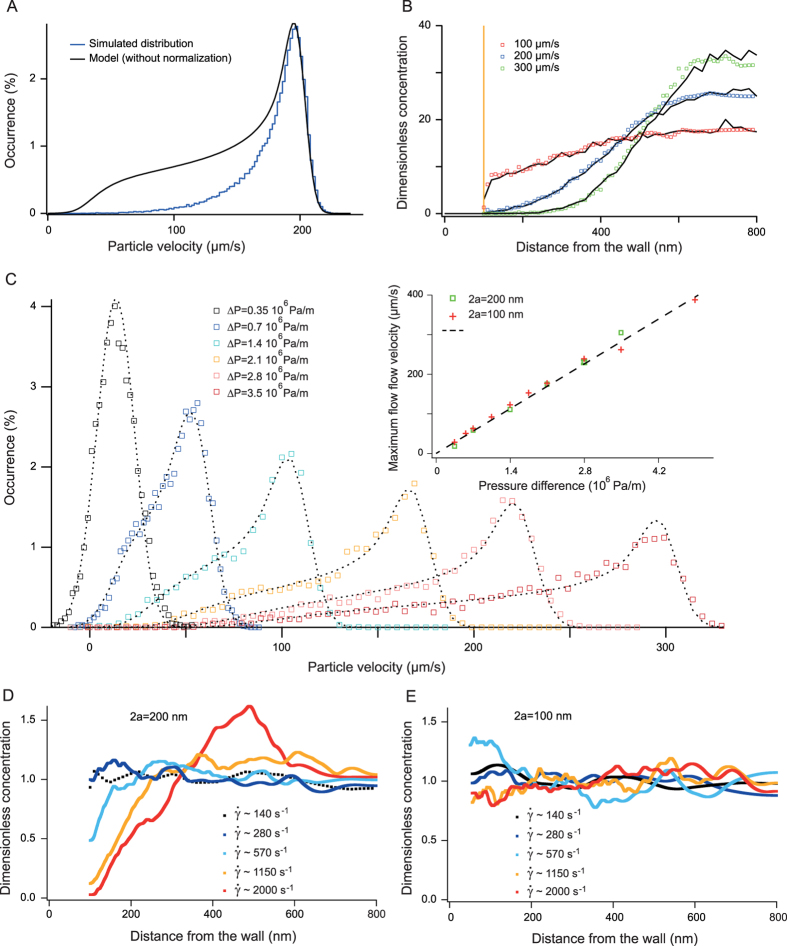
Measurement of particle concentration across the channel height in visco-elastic fluids. (**A**) The histogram represents the longitudinal velocity distribution obtained with a Brownian dynamics simulation including a centrifugal force field proportional to the square of the shear rate. The maximum flow velocity, channel height, and particle diameter have been set to 200 μm/s, 1.6 μm, and 200 nm, respectively. The solid black line corresponds to the velocity distribution expected from our model, showing that low velocity states are depleted. (**B**) The three black curves represent the repartition of tracers in the simulation across the channel half-height for *v*_*0*_ *= *100, 200, and 300 μm/s. The corresponding colored datasets are obtained by ratiometric analysis of longitudinal velocity distributions (see text). (**C**) Experimental velocity distributions were obtained with 2a *= *200 nm particles transported in a 1.6 μm thick channel. The fluid composed of 2% PVP 360 kDa was visco-elastic. The inset shows the linearity of the maximal velocity as a function of the pressure drop for 200 and 100 nm tracers (green and red datasets, respectively). (**C**-**D**) The plots show the repartition of tracers of 100 and 200 nm across the channel half-height for a range of pressure drops.
