# Supplementary material for: Metrology of confined flows using wide field nanoparticle velocimetry
Source: Sci Rep. 2015 May 14;5:10128. doi: 10.1038/srep10128 (PMC4431396; doi:10.1038/srep10128)

Metrology of confined flows using wide field nanoparticle velocimetry

Hubert Ranchon[1,2], Vincent Picot[1,2], Aurélien Bancaud[1,2]

[1] CNRS, LAAS, 7 avenue du colonel Roche, F-31400 Toulouse, France

[2] Univ de Toulouse, LAAS, F-31400 Toulouse, France

Supplemental Material

Supplementary Figure S1: Hydrodynamic model of particle transport. (A) The panel on the left shows the velocity profile along the z-direction for tracers of different sizes, as indicated in the legend. The black curve corresponds to the parabolic Poiseuille profile. The green curves are based on Fàxen model, *i.e.* neglecting HI, whereas the red velocity profiles are deduced from the work of Pasol et *al*. ([11], left panel). Note HI tend to smooth the velocity profile near the wall. On the right panel, the plot represents the corresponding velocity distributions with no fluctuations. **(B)** The graph shows three longitudinal velocity distributions with fluctuations for v_0=100, 200, 300 µm/s ignoring or considering HI (black and blue curves, respectively). The particle radius, channel height, diffusion coefficient, and inter-frame interval are set to a=100 nm, $2h$=1.6 µm, D_{lat}=0.2 µm^2/s, and τ=7 ms, respectively.

Supplementary Figure S2: Velocity distributions obtained from Brownian dynamics simulations. (A) The data points in the two graphs represent the output of Brownian dynamics simulations of random walkers transported in confined channels (see methods section in main text), and the corresponding fits are deduced from the model described in the main text. The parameters of the simulation are indicated in the inset. **(B)** The same simulation as in (A) is carried out for a range of inter-frame intervals of τ=5, 10, 20, 50 ms. Note that the shear rate is 500 s^{-1}. The lateral velocity distribution expectedly spreads over time, as shown in the graph on the left. Our model fails to reproduce the shape of longitudinal velocity histograms in the low velocity regime for τ>20 ms. **(C)** We investigated the precision in height and velocity measurements for different channel height spanning 600 to 3000 nm and inter-frame intervals spanning 1 to 16 ms (columns and lines in the table on the left, respectively). In every simulation, the particle diameter, viscosity and shear rate were set to 200 nm, 5.8 mPa.s, and 1000 s^{-1}. The Peclet number is roughly equal to 15, as shown in the

panel on the right. The fitting precision on the velocity is better than 1% (not shown), and that on the height is less than 5%. This analysis shows that the key parameter to characterize confined flows is the Peclet number rather than the criterion $\tau<2/\dot{\gamma}$. **(D)** We conducted a Brownian dynamics simulations using a fixed inter-frame interval of 4 ms, for two particle sizes of 2a=200 and 100 nm at a fixed level of confinement of 0.2, and gradually increasing the shear rate from 400 s^{-1} to 3200 s^{-1}. The viscosity is adjusted by a factor of 4 in order to set the viscosity above the threshold of Pe>4 (see Eq. (4)). The precision of the measurement of the velocity (datasets) and height (solid lines) has been estimated. This graph confirms that the precision of these measurements is better than 5% even if $\tau<2/\dot{\gamma}$.

Supplementary Figure S3: Chip fabrication and microscopy imaging. (A) Process flow for silicon chip fabrication and assembly on glass slides of 170 µm in thickness. Scanning electron micrographs, which show the lateral walls of the channels, were obtained at 2 kV (S-4800, Hitachi). **(B)** Imaging was performed with a Zeiss epifluorescence microscope equipped with the 38HE filter set (Zeiss), and with a Lumencor Light Engine emitting at 475 nm with a 28 nm bandwidth. An ANDOR iXon-885 camera was used using a binning of 2*2, and a pixel size of 103 nm. Pressure was monitored with a Fluigent Flowell controller delivering 1 bar.

Supplementary Figure S4: Characterization of particle size distribution. (A) Carboxylated polystyrene tracers of nominal diameter 200 nm and 100 nm were purchased from Bangs Labs and Invitrogen, respectively. They were characterized by single particle tracking in bulk, Dynamic Light Scattering (DLS, Malvern Instrument), and scanning electron microscopy, showing consistent average radius of 104+/-5, 101+/-4, and 104+/-3 nm, respectively, for 200 nm tracers. Note that the aspect ratio of these particles, as defined by the ratio of the largest to the smallest diameter was ~1.02, as inferred from the analysis of

electron micrographs. **(B)** Bulk rheological measurements of 2% PVP solution (MW 360 kDa). The graph shows the elastic modulus G' (red dataset) and loss modulus G'' (green dataset) as a function of the oscillatory frequency. The black solid lines correspond to the fit of the data with the Maxwell model with relaxation time of 10 ms. The inset represents the steady shear viscosity as a function of the shear rate.

Figure S1

Ranchon et al., 2014

A- Velocity profile for different levels of confinement (expression from Pasol *et al.,* 2005)

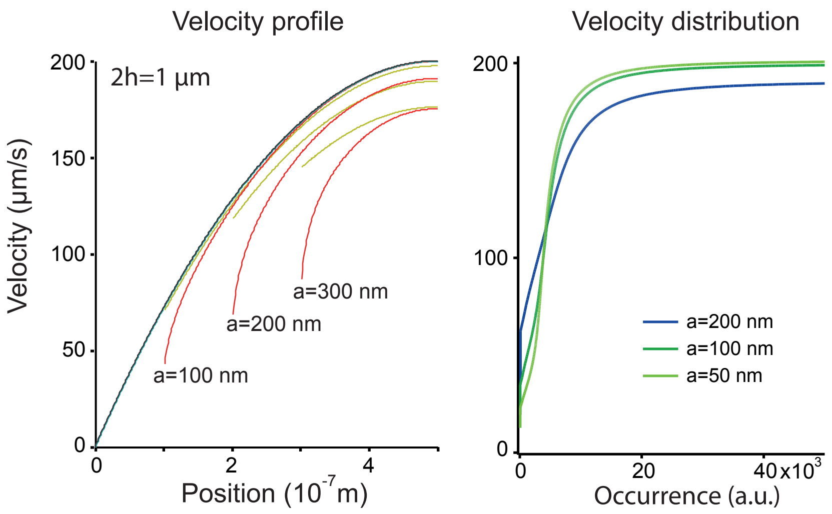

B- Convolution with the Gaussian noise extracted from the lateral velocity distribution

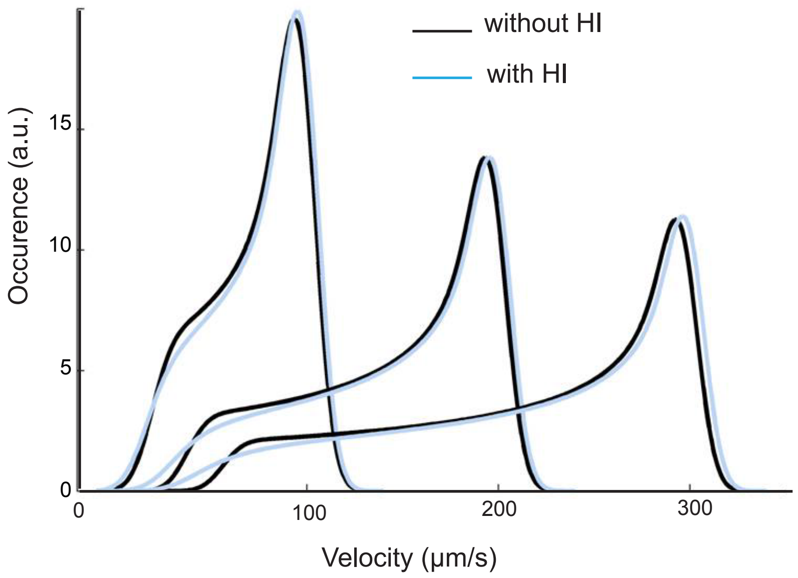

A Output of Brownian dynamics simulations (see method section in main text)

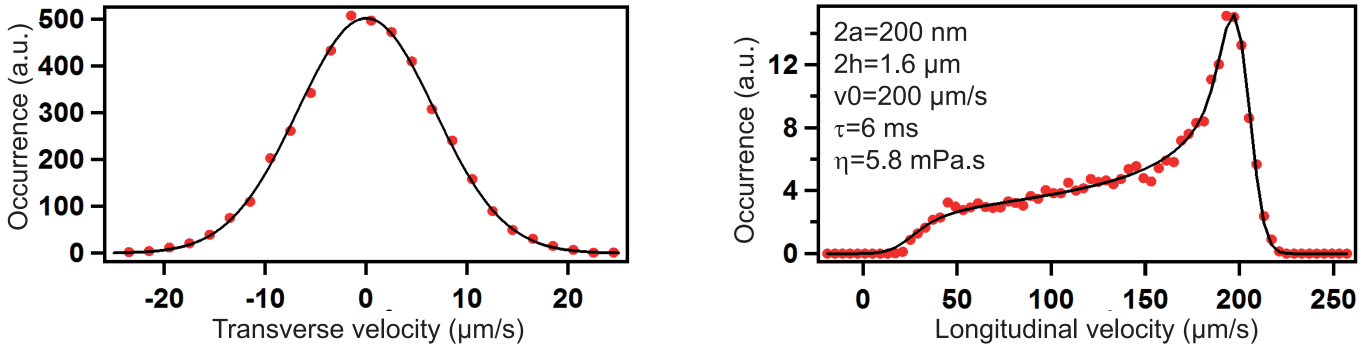

B- Effect of time sampling on fitting

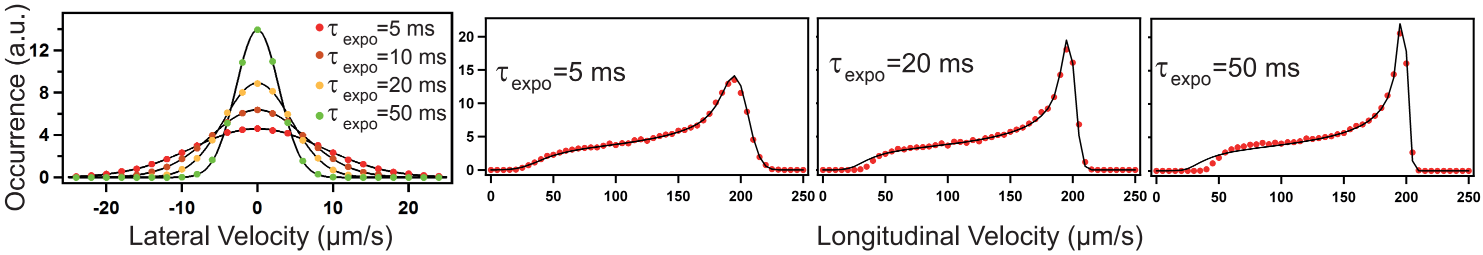

C- Effect of time sampling on fitting precision for a given shear rate of 1000 s-1

a=200 nm ; η = 5.8 mPa.s

Relative error in height measurement (%)

τ_{expo} (ms) \ a/h	0.33	0.2	0.125	0.1	0.067
1	8.3	8	6.3	2	1.3
2	5	4	3.8	6	8
4	1.7	4	6.3	-2	-2
8	0	2	-0.6	4	2
16	-3.3	2	-2.5	4	4

Peclet number

	0.33	0.2	0.125	0.1	0.067
1	16.1	14.7	14.7	13.6	14.1
2	17.5	16.7	15.7	15.7	15.1
4	18.6	17.4	16.6	15.925	15.5
8	19.2	18	17.1	17	17.5
16	19.1	18.4	17.2	16.375	16.5

D- Effect of shear rate on fitting precision for a given time interval of 4 ms

a=200 nm ; η = 4 mPa.s ; 2h= 1 µm (blue datasets)

a=100 nm ; η = 16 mPa.s ; 2h= 0.5 µm (green datasets)

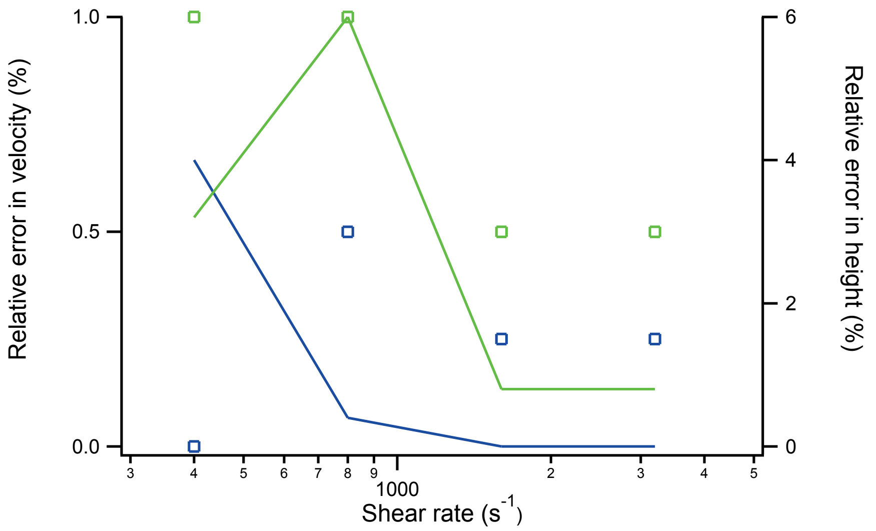

A- Process flow for channel fabrication

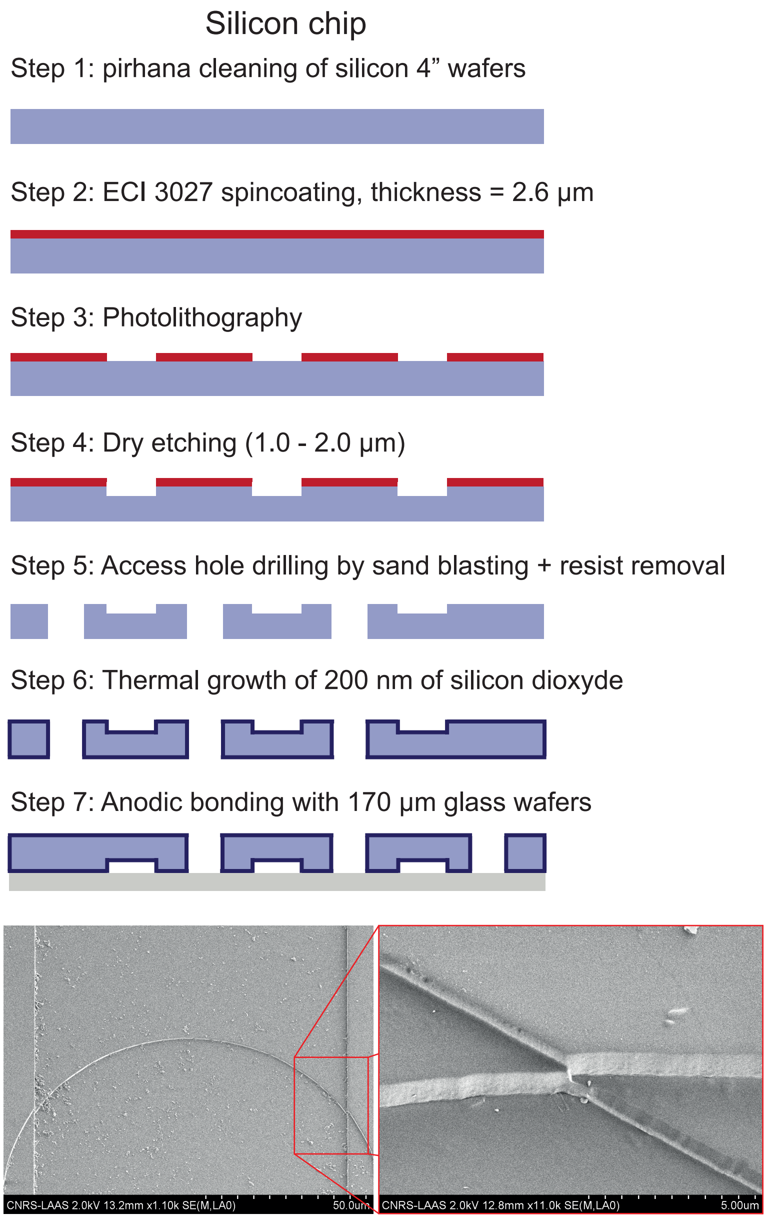

B- Imaging system

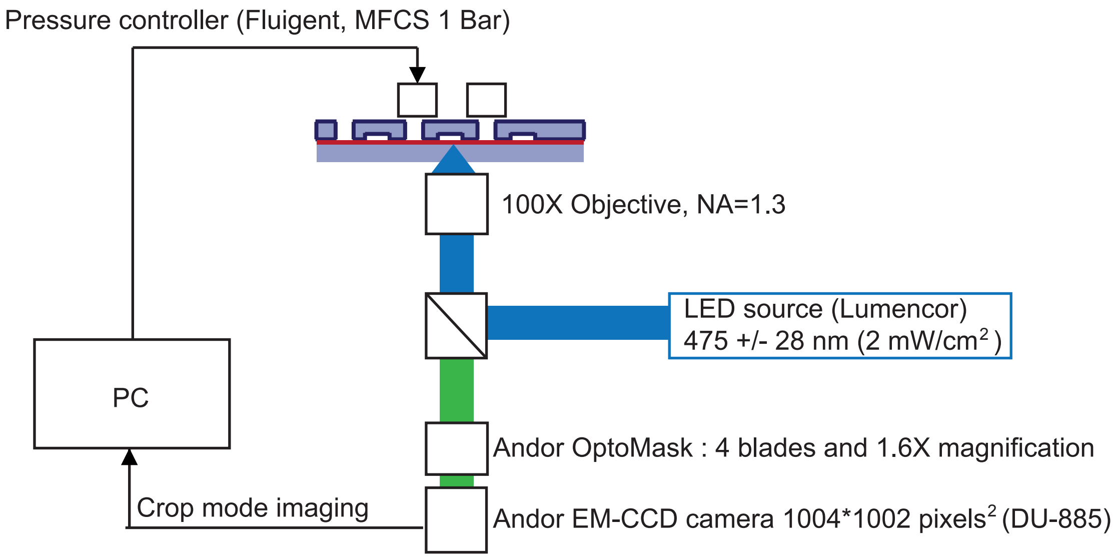

A- Characterization of nanoparticle size by electron microscopy, DLS, and particle tracking

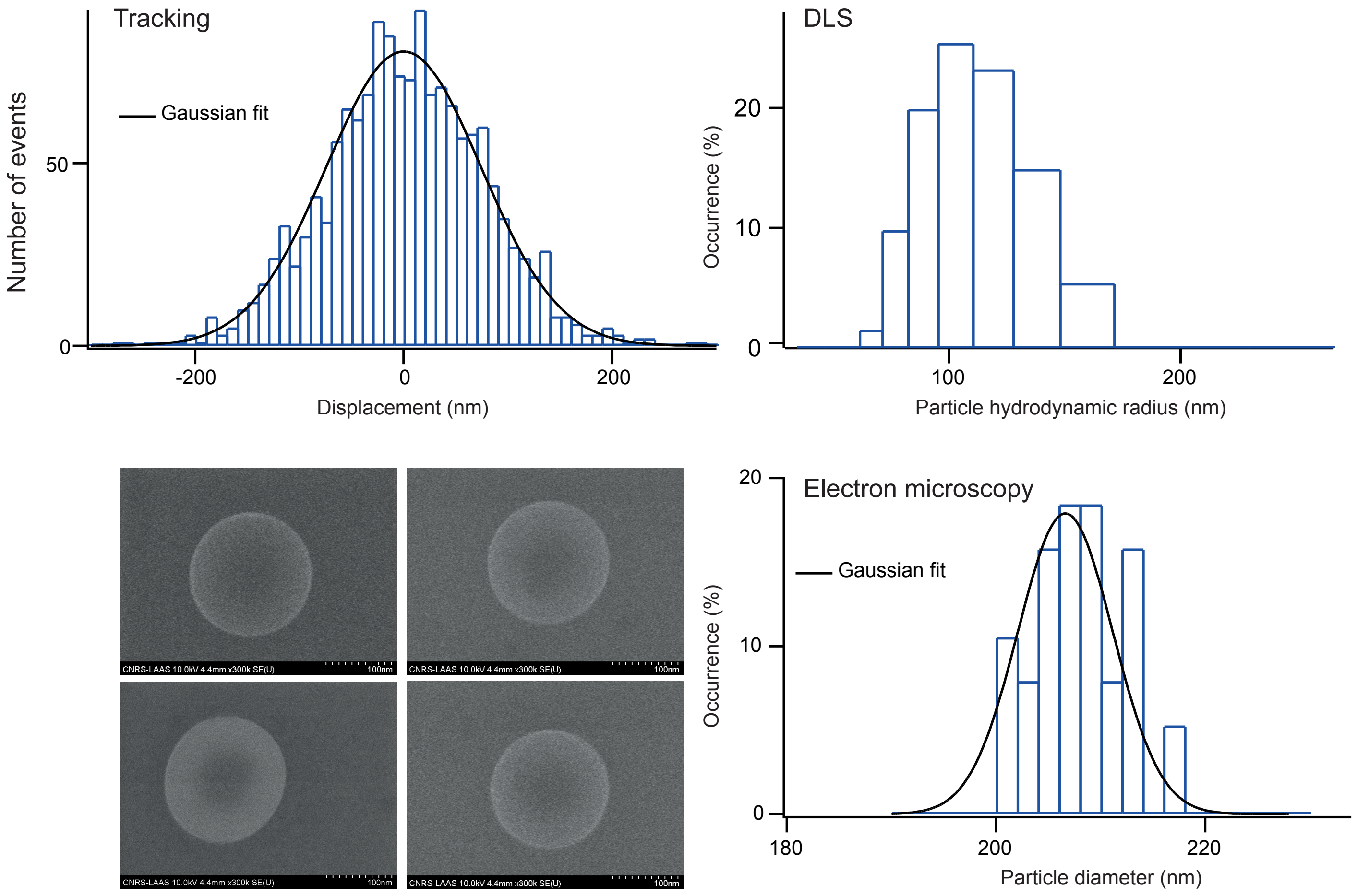

B- Rheological characterization of 2% PVP solution (MW 360 kDa)

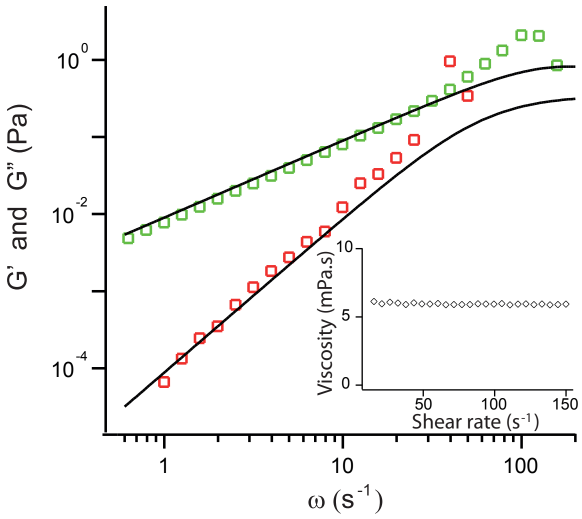

Supplement: Supplementary Information [file srep10128-s1.pdf]
